# Ovarian cancer treatment with a tumor-targeting and gene expression-controllable lipoplex

**DOI:** 10.1038/srep23764

**Published:** 2016-03-30

**Authors:** Zhi-Yao He, Feng Deng, Xia-Wei Wei, Cui-Cui Ma, Min Luo, Ping Zhang, Ya-Xiong Sang, Xiao Liang, Li Liu, Han-Xiao Qin, Ya-Li Shen, Ting Liu, Yan-Tong Liu, Wei Wang, Yan-Jun Wen, Xia Zhao, Xiao-Ning Zhang, Zhi-Yong Qian, Yu-Quan Wei

**Affiliations:** 1 Lab of Aging Research, State Key Laboratory of Biotherapy and Cancer Center, West China Hospital, Sichuan University and Collaborative Innovation Center of Biotherapy, Chengdu, Sichuan 610041, China; 2 Department of Gynecology and Obstetrics, West China Second Hospital, Sichuan University, Chengdu, Sichuan 610041, China; 3 The College of Life Science, Sichuan University, Chengdu, Sichuan 610041, China; 4 Department of Abdominal Oncology, Cancer Center, West China Hospital, Sichuan University, Chengdu, Sichuan 610041, China; 5 Department of Pharmacology and Pharmaceutical Sciences, School of Medicine, Tsinghua University and Collaborative Innovation Center of Biotherapy, Beijing 100084, China

## Abstract

Overexpression of folate receptor alpha (FRα) and high telomerase activity are considered to be the characteristics of ovarian cancers. In this study, we developed FRα-targeted lipoplexes loaded with an hTERT promoter-regulated plasmid that encodes a matrix protein (MP) of the vesicular stomatitis virus, F-LP/pMP_(2.5)_, for application in ovarian cancer treatment. We first characterized the pharmaceutical properties of F-LP/pMP_(2.5)_. The efficient expression of the MP-driven hTERT promoter in SKOV-3 cells was determined after an *in-vitro* transfection assay, which was significantly increased compared with a non-modified LP/pMP_(2.5)_ group. F-LP/pMP_(2.5)_ treatment significantly inhibited the growth of tumors and extended the survival of mice in a SKOV-3 tumor model compared with other groups. Such an anti-tumor effect was due to the increased expression of MP in tumor tissue, which led to the induction of tumor cell apoptosis, inhibition of tumor cell proliferation and suppression of tumor angiogenesis. Furthermore, a preliminary safety evaluation demonstrated a good safety profile of F-LP/pMP_(2.5)_ as a gene therapy agent. Therefore, FRα-targeted lipoplexes with therapeutic gene expression regulated by an hTERT promoter might be a promising gene therapy agent and a potential translational candidate for the clinical treatment of ovarian cancer.

Ovarian cancer is the leading cause of death from gynecologic malignancies worldwide^1^,^2^,^3^,^4^. Detection of early stages of ovarian cancer remains difficult and almost 90% of patients are diagnosed at an advanced stage (Stage III/IV, with widely metastatic disease within the peritoneal cavity) when prognosis is poor^5^,^6^. Metastasis of ovarian cancer includes the detachment of cancer cells and clusters from primary tumors, metastasis to the peritoneum and omentum (mesothelium), and the development of numerous tumor nodules in the mesothelium^7^,^8^. Therefore, although surgical procedures could be used to remove the bulk of tumor nodules, a complete resection is extremely hard to achieve^7^,^9^. The standard chemotherapy procedure involving several cytotoxic drugs has been established and optimized for ovarian cancer treatment (such as the combination of taxane and platinum); however, the overall cure rate has remained at approximately 30% for the last 20 to 30 years^7^,^10^,^11^,^12^. Therefore, for decades, considerable efforts have been devoted to developing more effective anti-tumor agents and better tumor-targeting strategies; and, in fact, several newly developed tumor-targeting therapeutic agents have already entered clinical trials^13^,^14^,^15^,^16^.

The metastasis of ovarian cancer is generally confined to the abdominal cavity, which has made ovarian cancer a good candidate for local gene therapy *via* intraperitoneal administration^17^,^18^. Indeed, ovarian cancer has been an important target in the field of human gene therapy over the past 20 years^19^,^20^, and we are developing such a potential therapy. The human telomerase reverse transcriptase (hTERT) promoter is a tumor-specific promoter that could preferentially direct target gene expression in human ovarian cancer cells that possess high telomerase activity^21^,^22^,^23^. Also, matrix protein (MP) from the vesicular stomatitis virus (VSV) exerts an antitumor effect by inducing tumor cell apoptosis, and has therefore been used in cancer gene therapy^24^,^25^,^26^,^27^,^28^. In this study, the hTERT promoter was used to drive the expression of MP in ovarian cancer cells, and was expected to enhance the specificity in gene therapy. Furthermore, the safety and efficiency of ovarian cancer gene therapy has been restricted due to the lack of tumor cell-targeting gene delivery systems^29^,^30^,^31^. Folate receptor alpha (FRα) is overexpressed in about 90% of ovarian cancers and is considered to be a good target for both drug design and gene delivery, and some FRα-targeted candidate drugs have already entered clinical trials for ovarian cancer therapy^32^,^33^,^34^,^35^. However, there are few clinical trials developing FRα-targeted lipoplexes for ovarian cancer gene therapy. In this study, we assessed FRα-targeted gene therapy as a novel treatment for ovarian cancer, which overexpressed FRα and also showed the hTERT promoter activity.

Herein, we developed a tumor-targeting gene delivery system based upon FRα-targeted lipoplexes and that includes a vector with a tumor-specific promoter (hTERT) to encode a gene (pMP) for ovarian cancer treatment. It is hypothesized that the FRα-targeted lipoplexes would deliver the pMP gene to the tumor site in an active fashion and drive the specific expression of the pMP gene by the hTERT promoter in ovarian cancer cells. The pharmaceutical properties, *in-vitro* biological activities, *in-vivo* antitumor effects, antitumor mechanisms, and preliminary toxicity evaluation of FRα-targeted lipoplexes loaded with pMP are presented in this report.

## Results

### Pharmaceutical properties of F-LP/pMP_(2.5)_

**Figure 1:**
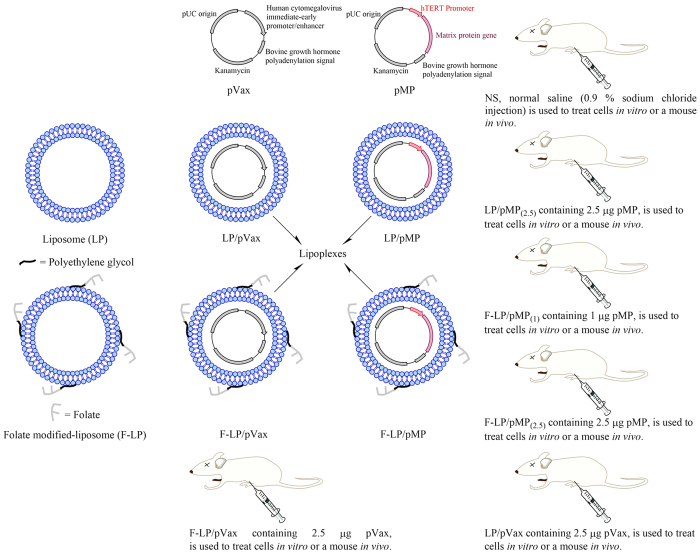
A schematic diagram illustrating the composition and differences among lipoplexes. LP, liposome; F-LP, folate-modified liposome; pVax, pVAX1^TM^ plasmid vector was purchased from Life Technologies Corporation (Frederick, MD, U.S.); pMP, recombinant pVAX1^TM^ plasmid encoded the human telomerase reverse transcriptase (hTERT) promoter and the gene for the matrix protein of the vesicular stomatitis virus; LP/pVax was composed of the LP and pVax; F-LP/pVax was composed of the F-LP and pVax; LP/pMP was composed of the LP and pMP; F-LP/pMP was composed of the F-LP and pMP.

**Figure 2:**
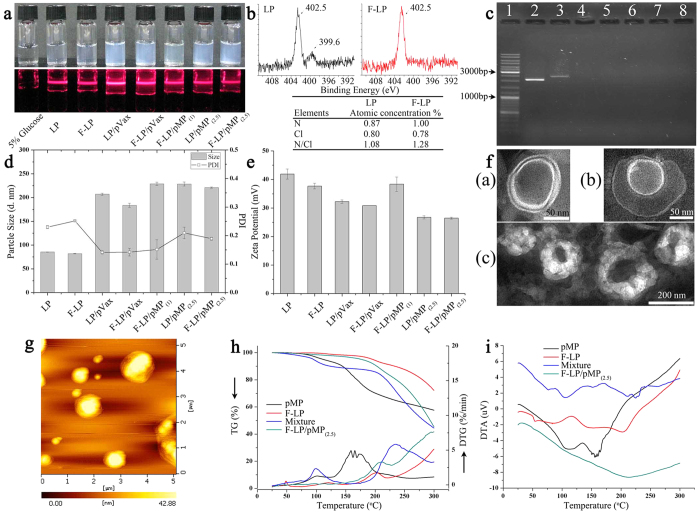
Pharmaceutical properties of F-LP/pMP_(2.5)_. (**a**) The appearance and Tyndall phenomenon of liposomes (LP & F-LP) and lipoplexes (LP/pVax, F-LP/pVax, F-LP/pMP_(1)_, LP/pMP_(2.5)_ & F-LP/pMP_(2.5)_). (**b**) XPS N_1s_ peak of LP & F-LP and quantification analysis for N atomic concentration on the surface of LP & F-LP. (**c**) Agarose gel electrophoresis of naked plasmid DNA and lipoplexes. Lane 1, DNA marker; lanes 2 & 3, respectively, naked pVax & pMP; lane 4, LP/pVax; lane 5, F-LP/pVax; lane 6, F-LP/pMP_(1)_; lane 7, LP/pMP_(2.5)_; lane 8, F-LP/pMP_(2.5)_. pVax or pMP was incorporated into LP or F-LP completely and lipoplexes were prepared with no free DNA. (**d**) Particle size and PDI of liposomes and lipoplexes (mean ± SD, n = 3). (**e**) Zeta potential of liposomes and lipoplexes (mean ± SD, n = 3). (**f**) TEM images of F-LP (**a**, bar = 50 nm) and F-LP/pMP_(2.5)_ (**b**, bar = 50 nm) & (**c**, bar = 200 nm). (**g**) AFM image of F-LP/pMP_(2.5)_. (**h**) TG and DTG curves of F-LP/pMP_(2.5)_. (i) DTA curve of F-LP/pMP_(2.5)_.

In this study we characterized the pharmaceutical properties of lipoplexes of 2 different liposomes. LP is an unmodified liposome and F-LP is a liposome modified by the addition of folate via polyethylene glycol as shown in Fig. 1. The lipoplexes incorporate an empty vector plasmid (pVax) as a control or a plasmid containing the therapeutic agent (pMP). The differences and relationships between liposomes and lipoplexes are illustrated in Fig. 1. Both liposomes and lipoplexes were clear colloids and displayed Tyndall effects as shown in Fig. 2a, indicating that the solutions of liposomes and lipoplexes were nanoscale colloidal solutions. To confirm the modification of liposomes with folate at the surface, we determined the nitrogen (N) atomic concentration (percentage) on the surface of F-LP and observed that it was higher than that for LP according to the quantification analysis with X-ray photoelectron spectroscopy (XPS) (Fig. 2b). The results implied the surface location of folate in F-LP and its potential capability for folate receptor-targeted delivery. Liposomes, after the incubation process, were capable of incorporating plasmid effectively so as to formulate lipoplexes as determined by gel electrophoresis (Fig. 2c; Lanes 4 to 8). The particle sizes of liposomes and lipoplexes were about 80 nm and 180–230 nm, respectively (Fig. 2d). The zeta potentials of liposomes and lipoplexes were 27–42 mV as shown in Fig. 2e. Liposomes and lipoplexes showed the typical lipid bilayer morphology under transmission electron microscopy (TEM; Fig. 2f[a,b], respectively). The size of F-LP/pMP_(2.5)_ was about 200 nm as shown in Fig. 2f(c) and coincided with the results of particle size measurement. Figure 2g also shows the spheroidal morphologic characteristics of F-LP/pMP_(2.5)_ under atomic force microscopy (AFM). The results of thermogravimetric (TG), derivative thermogravimetric (DTG), and differential thermal analysis (DTA) indicated that the thermal properties of F-LP/pMP_(2.5)_ were different from that of pMP, F-LP and their physical mixtures (Fig. 2h,i). To summarize, F-LP/pMP_(2.5)_ was prepared as a nanoscale spheroidal particle with a bilayer structure, with its own physical and chemical properties.

### F-LP-mediated gene expression and the biologic activity of MP

**Figure 3:**
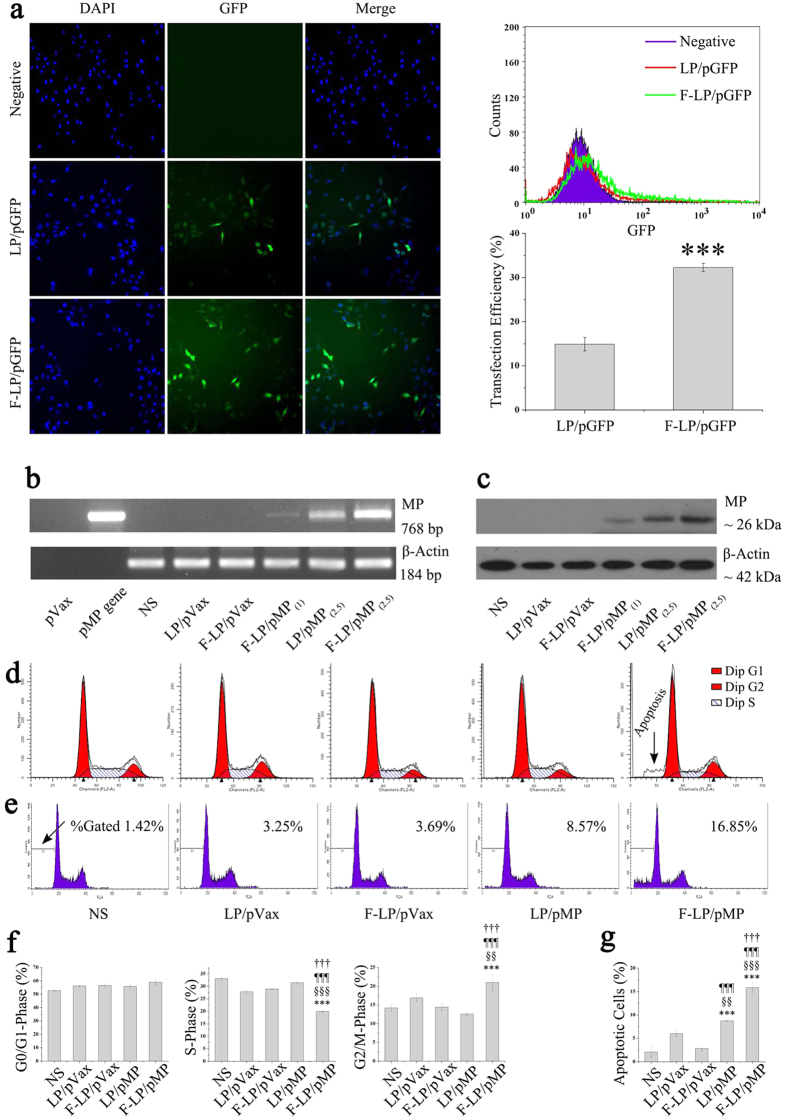
*In-vitro* F-LP-mediated gene expression and biologic activity of MP in SKOV-3 cells. (**a**) F-LP/pGFP-mediated reporter gene expression, including fluorescence photographs (original magnification, ×200), flow cytometric analysis and statistical data (mean ± SD, n = 3, ^***^P < 0.001, F-LP/pGFP *versus* LP/pGFP). (**b**) Expression of MP at the mRNA level determined by RT-PCR (naked pVax and pMP plasmids did not express β-actin). (**c**) Expression of MP at the protein expression level was determined by western blot analysis. (**d**) Cell cycle analysis by flow cytometry. (**e**) Apoptosis analysis by flow cytometry. (**f**) The effect of MP on cell cycle (NS indicates that the cells were treated with normal saline (mean ± SD, n = 3, ^***^P < 0.001, NS *versus* LP/pVax, F-LP/pVax, LP/pMP and F-LP/pMP; ^§§^*P* < 0.01, ^§§§^*P* < 0.001, LP/pVax *versus* F-LP/pVax, LP/pMP and F-LP/pMP; ^¶¶¶^*P* < 0.001, F-LP/pVax *versus* LP/pMP and F-LP/pMP; ^†††^*P* < 0.001, LP/pMP *versus* F-LP/pMP). (**g**) MP-induced cellular apoptosis (mean ± SD, n = 3, ^***^P < 0.001, NS *versus* LP/pVax, F-LP/pVax, LP/pMP and F-LP/pMP; ^§§^*P* < 0.01, ^§§§^*P* < 0.001, LP/pVax *versus* F-LP/pVax, LP/pMP and F-LP/pMP; ^¶¶¶^*P* < 0.001, F-LP/pVax *versus* LP/pMP and F-LP/pMP; ^†††^*P* < 0.001, LP/pMP *versus* F-LP/pMP).

In the *in-vitro* cell transfection assay, folate-modified F-LP achieved significantly higher GFP transfection efficiency than did LP in the FRα-positive SKOV-3 cells (95% of the cells express FRα, Supplementary Fig. S1a; Fig. 3a; *P* < 0.001). Although A2780 cells expressed fewer FRα (5%, Supplementary Fig. S1b), there was no significant difference between the transfection efficiencies of F-LP and LP (Supplementary Fig. S1c; *P* > 0.05), which indicated that FRα-targeted liposomes (F-LP) could specifically increase the transfection efficiency in FRα-positive cells. RT-PCR and western blots demonstrated that the hTERT promoter could drive cell-specific expression of MP at both the mRNA level and protein levels, and that F-LP/pMP_(2.5)_ manifested more MP expression than did LP/pMP_(2.5)_ in SKOV-3 cells (Fig. 3b,c). Furthermore, cell cycle analysis showed that F-LP/MP transfection was able to increase the number of phase G2/M cells (*P* < 0.01) and decrease the number of phase S cells (*P* < 0.001) (Fig. 3d,f). F-LP/pMP- transfected SKOV-3 cells exhibited significantly more apoptosis than LP/pMP-treated cells (Fig. 3e,g; *P* < 0.001). In summary, F-LP exhibited a higher transfection efficiency toward folate receptor-positive cells *in vitro* compared with LP; improving both the expression of the reporter gene and the therapeutic gene. This also contributed to an enhanced biologic activity of MP in ovarian cancer cells by showing a significant increase in cellular apoptosis after treatment of F-LP/MP.

### Antitumor study and MP gene expression in tumor tissues

**Figure 4:**
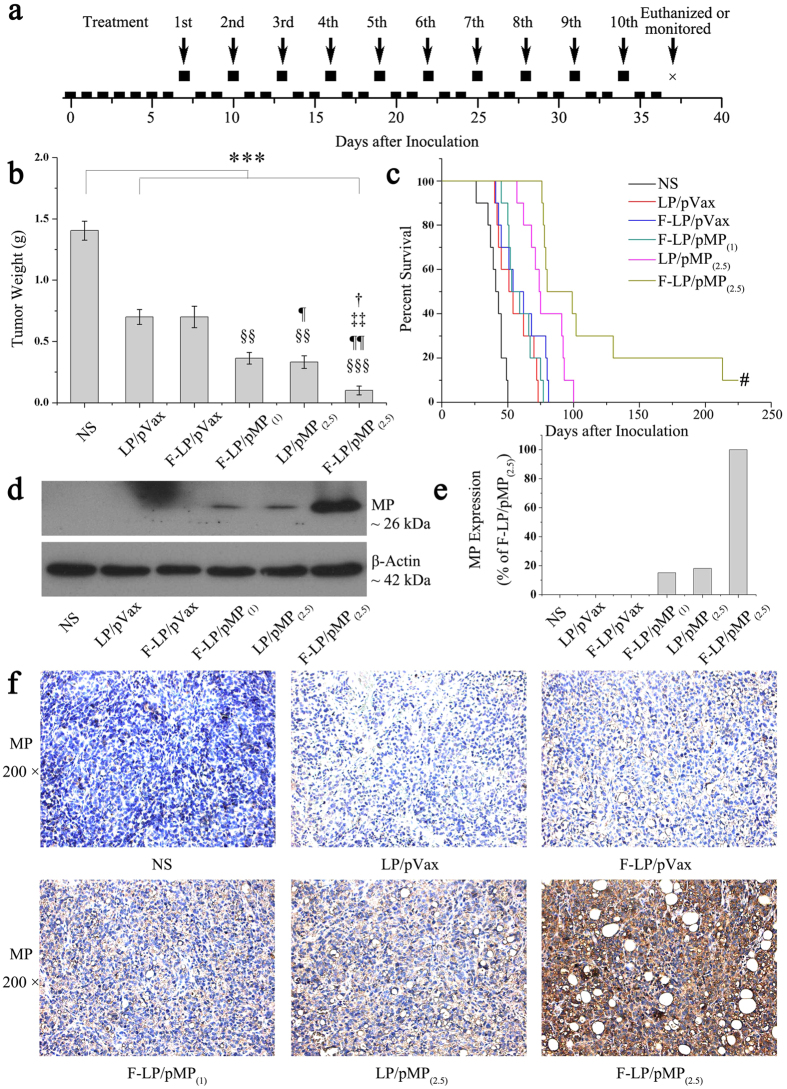
*In-vivo* antitumor effects of F-LP/pMP_(2.5)_ and gene expression for MP in tumor tissues. (**a**) The dosing regimen for ovarian cancer gene therapy (the boxes represent days, so that raised boxes are days of treatment and boxes on the timeline are days without treatment; on the 37^th^ day after inoculation, mice were euthanized for the antitumor study or monitored continuously for survival study). (**b**) The mean tumor weight of peritoneal ovarian cancer metastasis (mean ± SE, n = 10, ^***^P < 0.001, NS *versus* LP/pVax, F-LP/pVax, F-LP/pMP_(1)_, LP/pMP_(2.5)_ and F-LP/pMP_(2.5)_; ^§§^*P* < 0.01, ^§§§^*P* < 0.001, LP/pVax *versus* F-LP/pVax, F-LP/pMP_(1)_, LP/pMP_(2.5)_ and F-LP/pMP_(2.5)_; ^¶^*P* < 0.05, ^¶¶^*P* < 0.01, F-LP/pVax *versus* F-LP/pMP_(1)_, LP/pMP_(2.5)_ and F-LP/pMP_(2.5)_; ^‡‡^*P* < 0.01, F-LP/pMP_(1)_ *versus* F-LP/pMP_(2.5)_; ^†^*P* < 0.05, LP/pMP_(2.5)_ *versus* F-LP/pMP_(2.5)_). (**c**) Survival of mice bearing tumors (#, the cured mouse was euthanatized on the 550^th^ day after inoculation). (**d**) Expression levels of MP in tumor tissues using western blot analysis. (**e**) The relative expression levels of MP in different experimental groups using western blot analysis (compared with the F-LP/pMP_(2.5)_ group). (**f**) Expression of MP in tumor tissues with immunohistochemical staining (original magnification, ×200). (**d–f**) were representative results from three mice.

LP- and F-LP- based lipoplexes loaded with pVax and pMP were applied to treat a SKOV-3 ovarian cancer model in nude mice *via* intraperitoneal injection and the treatment regimen was shown in Fig. 4a. After 10 treatments, F-LP/pMP_(2.5)_ showed dramatic anti-tumor effects compared with other groups. F-LP/pMP_(2.5)_ inhibited tumor growth significantly more than other lipoplexes (LP/pVax, ^§§§^*P* < 0.001; F-LP/pVax, ^¶¶^*P* < 0.01; F-LP/pMP_(1)_, ^‡‡^*P* < 0.01; LP/pMP_(2.5)_, ^†^*P* < 0.05) (Fig. 4b), and prolonged median survival of mice to 80 days, which was significantly greater than the 74 days (LP/pMP_(2.5)_), 53 days (F-LP/pMP_(1)_), 54 days (F-LP/pVax), 51 days (LP/pVax) or 41 days (NS) in the other groups, respectively (log-rank, *P* < 0.001) (Fig. 4c). Furthermore, one mouse was cured after the treatment with F-LP/pMP_(2.5)_. The cured mouse was euthanatized on the 550^th^ day after inoculation and no tumor nodule was found in its abdominal cavity. Such anti-tumor effects of F-LP/pMP_(2.5)_ could be explained by the dramatically augmented MP expression in tumors after treatment with F-LP/pMP_(2.5)_ (Fig. 4d,e). The F-LP/pMP_(2.5)_-treated tumor tissue also displayed strong MP expression by immunohistochemistry (Fig. 4f). This might be due to the folate receptor-targeted properties of F-LP/pMP_(2.5),_ which thus achieved a more specific and stronger expression of pMP in tumor cells compared with other lipoplexes. When it came to the other groups of lipoplexes, mice treated with LP/pMP_(2.5)_ also achieved a longer survival than the F-LP/pMP_(1)_, LP/pVax or F-LP/pVax group, which was supported by the increased expression of MP in tumors in the LP/pMP_(2.5)_-treated group (Fig. 4d,e). Therefore, our results suggested that F-LP/pMP_(2.5)_ effectively enhanced the MP expression in tumor tissues, inhibited tumor growth, and prolonged the survival of tumor-bearing mice.

LP/pVax and F-LP/pVax displayed antitumor effects compared with NS (Fig. 4b, ***P < 0.001). Theoretically, the pVax empty vector should not show antitumor effects *in vivo*. Cationic liposomes injected intraperitoneally are at high peritoneal concentrations and are retained for more than 48 h^36^, thus enhancing their antitumor activity (including that of F-LP/pVax and LP/pVax). Because the abdominal tumors were treated by intraperitoneal injection, controls (F-LP/pVax and LP/pVax) had a direct effect on growth and the (micro)environment of tumors and then inhibited tumor growth. Finally, controls (F-LP/pVax and LP/pVax) displayed antitumor effects compared to NS (Fig. 4b). Also, the antitumor effect of controls might be attributed to adaptive cellular immunity elicited by cationic liposome: pVax complexes^37^. However, compared with controls (F-LP/pVax and LP/pVax), the groups with the pMP gene still showed stronger tumor growth inhibition (Fig. 4b), and F-LP/pMP prolonged the survival of mice significantly (Fig. 4c, *P* < 0.001). IHC staining and western blot analyses indicated that MP expression resulted in antitumor effects directly (Fig. 4d–f), and that therefore, pMP caused the specific antitumor effects *in vivo*.

### Mechanistic antitumor study

**Figure 5:**
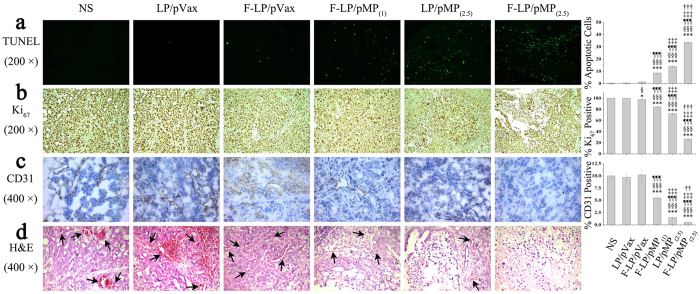
Antitumor mechanism of F-LP/pMP_(2.5)_. Effects of F-LP/pMP_(2.5)_ on cancer cell apoptosis (**a**) original magnification, ×200), proliferation (**b**) original magnification, ×200) and angiogenesis (**c**) original magnification, ×400) determined by TUNEL analysis, Ki_67_ and CD31 staining, respectively (mean ± SD, n = 10, ^*^P < 0.05, ^***^P < 0.001, NS *versus* LP/pVax, F-LP/pVax, F-LP/pMP_(1)_, LP/pMP_(2.5)_ and F-LP/pMP_(2.5)_; ^§^*P* < 0.05, ^§§§^*P* < 0.001, LP/pVax *versus* F-LP/pVax, F-LP/pMP_(1)_, LP/pMP_(2.5)_ and F-LP/pMP_(2.5)_; ^¶¶¶^*P* < 0.001, F-LP/pVax *versus* F-LP/pMP_(1)_, LP/pMP_(2.5)_ and F-LP/pMP_(2.5)_; ^‡‡‡^*P* < 0.001, F-LP/pMP_(1)_ *versus* F-LP/pMP_(2.5)_; ^††^*P* < 0.01, ^†††^*P* < 0.001, LP/pMP_(2.5)_ *versus* F-LP/pMP_(2.5)_). (**d**) H&E staining of tumor tissues (original magnification, ×400, arrows indicate blood vessels carrying erythrocytes).

The mechanisms underlying the antitumor effects of F-LP/pMP_(2.5)_ were studied by TUNEL, Ki_67_, CD31, and H&E staining as shown in Fig. 5. The results indicated that mice treated with F-LP/pMP_(2.5)_ showed a significant increase in apoptosis within tumor cell populations compared with other groups as determined by TUNEL assay. F-LP/pMP_(2.5)_ also significantly induced more cellular apoptosis compared to F-LP/pMP_(1)_ (^‡‡‡^*P* < 0.001) or LP/pMP_(2.5)_ (^†††^*P* < 0.001) as shown in Fig. 5a. The LP/pMP_(2.5)_ group, due to the higher dose of the pMP vector, additionally had more apoptotic cells compared with F-LP/pMP_(1)_ (^‡‡‡^*P* < 0.001).

The expression of MP also affected the proliferation of tumor cells. F-LP/pMP_(2.5)_ significantly inhibited cancer cell proliferation compared with other groups (F-LP/pMP_(1)_ [^‡‡‡^*P* < 0.001] and LP/pMP_(2.5)_ [^†††^*P* < 0.001] by Ki_67_ staining as shown in Fig. 5b). We also noted that F-LP/pVax had a weak anti-proliferative effect compared with the NS (^*^P < 0.05) or LP/pVax groups (^§^*P* < 0.05), and due to the lower dose of pMP, F-LP/pMP_(1)_, had a weaker anti-proliferative effect than did LP/pMP_(2.5)_ (^‡‡‡^*P* < 0.001).

Furthermore, the F-LP/pMP_(2.5)_ group also showed an anti-angiogenic effect in tumors compared to F-LP/pMP_(1)_ (^‡‡‡^*P* < 0.001) or LP/pMP_(2.5)_ (^††^*P* < 0.01) by CD31 staining (Fig. 5c). LP/pMP_(2.5),_ with more pMP loaded, inhibited angiogenesis more effectively than did F-LP/pMP_(1)_ (^‡‡‡^*P* < 0.001).

H&E staining of tumor sections suggested that there were more vessels, red blood cells and cancer cells in the tumors treated by NS, LP/pVax or F-LP/pVax. In contrast, tumor tissues treated with F-LP/pMP_(2.5)_, F-LP/pMP_(1)_, or LP/pMP_(2.5)_ showed fewer vessels and more dead cancer cells (Fig. 5d).

Thus, the results suggest that the antitumor effects of F-LP/pMP_(2.5)_ are achieved by inducing cancer cell apoptosis, inhibiting tumor cell proliferation, and suppressing tumor angiogenesis.

### Preliminary safety evaluation

**Figure 6:**
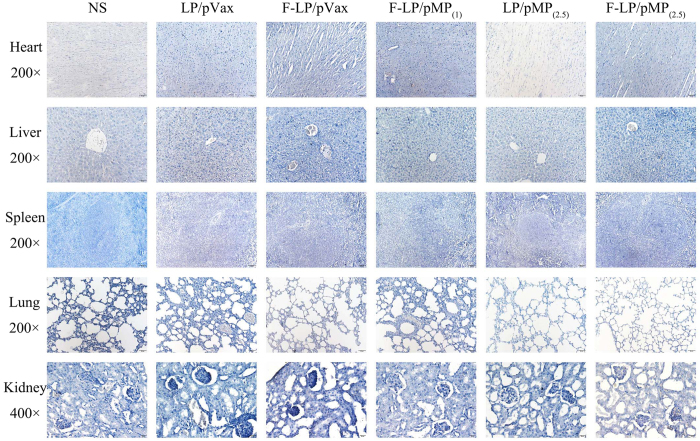
IHC for MP in non-cancerous tissues. The MP gene was not expressed in the non-cancerous tissues including heart, liver, spleen, lung (original magnification, ×200), or kidney (original magnification, ×400) after intraperitoneal injection.

We evaluated the safety of F-LP/pMP_(2.5)_ in female mice with H&E staining of vital organ sections and serologic biochemical analysis. IHC for MP in vital non-cancerous tissues was also performed. These tissues were not targeted and did not express MP (Fig. 6). We observed vacuolar degeneration of hepatocytes in the NS H&E sections (Supplementary Fig. S2); and inflammatory cells were found in the lung tissues (Supplementary Fig. S3). Compared with the NS group, F-LP/pMP_(2.5)_ ameliorated both the liver damage (Supplementary Fig. S2) and reduced the pulmonary inflammatory response (Supplementary Fig. S3) induced by the growth of peritoneal ovarian cancer. Vital organ sections all showed normal histologic morphologies in the F-LP/pMP_(2.5)_ group and little toxicity of F-LP/pMP_(2.5)_ was found (Supplementary Fig. S2–S4).

**Figure 7:**
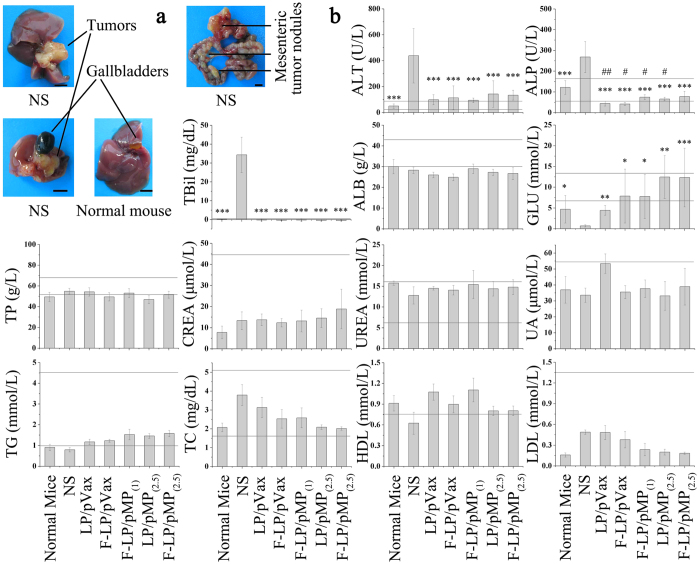
The typical distribution of tumor nodules and serologic biochemical analyses. a for images (scale bars = 0.5 centimeter) and b for the graphs (mean ± SD, n = 4–6, ^*^P < 0.05, ^**^P < 0.01, ^***^P < 0.001, NS *versus* normal mice, LP/pVax, F-LP/pVax, F-LP/pMP_(1)_, LP/pMP_(2.5)_ and F-LP/pMP_(2.5)_; ^#^*P* < 0.05, ^##^*P* < 0.01, normal mice *versus* LP/pVax, F-LP/pVax, F-LP/pMP_(1)_, LP/pMP_(2.5)_ and F-LP/pMP_(2.5)_). ALT, alanine aminotransferase; ALP, alkaline phosphatase; TBil, total bilirubin; ALB, albumin; GLU, glucose; TP, total protein; CREA, creatinine; UREA, urea; UA, uric acid; TG, triglycerides; TC, total cholesterol; HDL, high-density lipoprotein-cholesterol; LDL, low-density lipoprotein-cholesterol. The horizontal lines indicate the reference range for each analyte. The reference ranges for ALT, ALP, and TBil were 27–90 U/L, 57–160 U/L and 0.2–0.7 mg/dL, respectively. The reference ranges for ALB, GLU, TP, CREA, UREA and UA were 30–43 g/L, 7.38–13.44 mmol/L, 53–68 g/L, < 44.2 μmol/L, 6.07–16.07 mmol/L, and <54.13 μmol/L, respectively. The reference ranges for TG, TC, HDL and LDL were 1.00–4.55 mmol/L, 1.81–5.12 mg/dL, >0.78 mmol/L, and <1.35 mmol/L, respectively.

As shown in Fig. 7a, ovarian cancer cells metastasized to the liver in the NS group and tumor nodules were newly developed in the hepatic portal. This obviously affected liver function of mice in the NS group and resulted in abnormalities in corresponding biochemical indices (Fig. 7b, ALT, ALP, and TBil). Due to the strong anti-tumor effects of F-LP/pMP_(2.5)_, this treatment effectively protected the liver from the metastatic tumor cells and resulted in a maintenance of normal liver functions, which was illustrated by the similar normal liver biochemistry in F-LP/pMP_(2.5)_-treated mice. In addition, as mesenteric tumor nodules (Fig. 7a) in mice would cause intestinal obstruction and motility disorders and might affect the digestion and absorption of food, blood glucose levels (Fig. 7b, GLU) decreased significantly in mice in the NS group compared with normal mice (*P* < 0.05). In contrast, GLU returned to normal after gene therapy with pMP, which was expected to improve the quality of life for tumor-bearing mice. Moreover, F-LP/pMP_(2.5)_ did not cause abnormal fluctuations in TP levels; and renal functions maintained their normal level after gene therapy as reflected in CREA, UREA, and UA analyses (Fig. 7b). Blood lipid levels, including TG, TC, HDL, and LDL (Fig. 7b), were all similar to those of normal mice after treatment with F-LP/pMP_(2.5)_. Preliminary safety evaluations suggested that F-LP/pMP_(2.5)_ offered no toxicity during the animal tests and was safe to use in the gene therapy of ovarian cancer.

## Discussion

Tumor-targeted gene delivery systems enhance the specific delivery and expression of therapeutic genes at tumor sites and thus improve the efficacy of gene therapy^38^,^39^,^40^. Over 90% of human epithelial ovarian cancer tissues show overexpression of FRα, which is considered to be an ideal model for a targeted delivery system^33^. In the present study, we demonstrated a folate-modified gene delivery system for the treatment of ovarian cancer using SKOV-3 cells as a model. The folate-modified lipoplexes showed improved properties for targeted gene delivery both *in vitro* and *in vivo*, which contributed to the strong anti-tumor effects of F-LP-based lipoplexes. Such a conclusion is supported by our results, namely, that the folate-modified lipoplexes significantly increased gene transfection efficiencies of GFP in FRα-positive SKOV-3 cells *in vitro* as detected by flow cytometry. F-LP/pMP also increased the expression of MP in SKOV-3 cells compared with the LP/pMP group *in vitro* as detected by western blotting and PCR assay. Treatment of tumor-bearing mice with F-LP/pMP significantly inhibited tumor growth and prolonged survival of mice. Greatly increased expression of pMP was found in tumor tissue of mice treated with F-LP/pMP compared with other groups, such as the LP/pMP group; and this might be due to the FRα-targeting properties of F-LP carriers, which would result in the induction of tumor cell apoptosis, inhibition of tumor cell proliferation, and tumor angiogenesis as detected by immunohistochemical staining. The pVax lipoplexes showed an antitumor effect, which might be attributed to adaptive cellular immunity elicited by cationic lipid: plasmid DNA complexes^37^. However, the pVax lipoplexes could not prolong the survival so well as F-LP/pMP or LP/pMP. Thus, we conclude that the folate-modified- carrier-based gene delivery system has advantages in improving the effectiveness of gene expression in both FRα-positive tumor cells *in vitro* and *in-vivo* tumor tissues.

The oncolytic virus VSV has been used for human cancer gene therapy; however, its inherent neurotoxicity has prevented its further clinical application^41^,^42^. MP encoded by the VSV genome is the major antitumor component of VSV^41^,^43^. Although human cytomegalovirus (CMV) promoter-regulated plasmids expressing MP have been constructed and used in cancer gene therapy, expression of MP in normal cells induced cellular apoptosis and toxicity to normal tissues, and activated the immune system^41^,^44^,^45^. To deal with the potential safety issues surrounding the MP gene, we replaced the CMV promoter with a tumor-specific hTERT promoter in this study. The new plasmid (pMP) was constructed and MP was only expressed specifically in cancer cells, which could provide the required transcription factors (including c-Myc, SP1, human papillomavirus type 16 E6, and so on) and chromatin environment for the hTERT promoter^46^,^47^,^48^,^49^,^50^; as we observed the efficient expression of MP driven by the hTERT promoter in cancer cells in a cell transfection assay with F-LP/pMP *in vitro*. MP expression was determined by western blotting and PCR, and the MP-expressed cells underwent apoptosis and cell cycle dysfunction. The MP plasmid with the hTERT promoter also showed strong anti-tumor effects in SKOV3 tumor-bearing mice with few side effects. Mice were treated with LP/pVax, F-LP/pVax, LP/pMP, or F-LP/pMP (F-LP/pMP_(1)_ and F-LP/pMP_(2.5)_), and the expression of MP in tumor tissues was confirmed by western blot and IHC staining. All lipoplexes loaded with pMP exhibited considerable anti-tumor effects, and F-LP/pMP_(2.5)_ at a higher dose of pMP inhibited tumor growth more effectively and prolonged the median survival time of mice significantly compared with other groups (log-rank, *P* < 0.001). The efficient expression of pMP in tumors contributed to the induction of tumor cell apoptosis, inhibition of tumor cell proliferation, and suppression of tumor angiogenesis as determined by IHC. Even the higher dose of pMP delivery (F-LP/pMP_(2.5)_) did not cause any side effects in mice. The levels of ALT, ALP and TBil increased significantly in the mice of the NS group (*P* < 0.001), but these abnormally increased levels of ALT were downregulated after the mice were treated with lipoplexes. Furthermore, ALP was back to normal levels in the mice treated with F-LP/pMP_(2.5)_, which ameliorated the liver damage caused by ovarian cancer metastasis; and the level of ALP was then maintained^51^. The values of TBil, ALB, GLU, TP, and TG were similar to those of the normal mice and the levels of CREA, UREA, UA, TC, HDL, and LDL were also within the reference ranges after treatment with F-LP/pMP_(2.5)_^51^,^52^,^53^,^54^. The preliminary safety of F-LP/pMP_(2.5)_ was assessed, and no abnormalities were observed in vital organs of any pMP-treated groups of mice. Thus, our results showed that pMP with an hTERT promoter was a safe and efficient therapeutic vector in the gene therapy of ovarian cancer. We have additionally applied folate-modified carriers to pMP delivery in ovarian cancer treatment in the present study. The targeted delivery of pMP to tumor sites significantly improved the efficacy of its anti-tumor effects compared with LP/pMP. Therefore, we concluded that the novel F-LP/pMP_(2.5)_ lipoplexes were promising anticancer agents with good safety profiles, and that they might be potential candidates for clinical use in ovarian cancer treatments. This study was performed on SKOV-3 cells and the applicability in other ovarian cancer cells needs to be further explored.

In conclusion, we developed a novel and safe FRα-targeted lipoplex loaded with pMP using a tumor-specific hTERT promoter, F-LP/pMP_(2.5)_. The prepared lipoplexes are nanoscale spheroidal particles with bilayer structures, and appear as a clear solution with its own physicochemical properties. The lipoplexes were composed of F-LP as the vector carrier and pMP as the therapeutic gene. Folate-modified liposomes enhanced the uptake of F-LP/pMP_(2.5)_ by ovarian cancer cells due to the affinity of FRα and ligand F (folate) *in vitro*. Additionally, pMP, regulated by an hTERT promoter, was designed to be expressed specifically in cancer cells, inducing tumor cell apoptosis, while being safe to normal cells. F-LP/pMP_(2.5)_ was able to express MP both in tumor cells *in vitro* and in tumor tissues *in vivo*. The expression of MP in tumors led to an efficient anti-tumor effect of F-LP/pMP_(2.5)_ mediated by the induction of cancer cell apoptosis, inhibition of tumor cell proliferation, and suppression of tumor angiogenesis. The survival of mice treated with F-LP/pMP_(2.5)_ was prolonged significantly (log-rank *P* < 0.001). Preliminary safety evaluations implied that F-LP/pMP_(2.5)_ was a safe and efficient therapeutic agent for use in gene therapy of ovarian cancer. Therefore, the FRα-targeted lipoplex with therapeutic gene expression regulated by an hTERT promoter might be a promising candidate for clinical treatment of ovarian cancer.

## Methods

### Materials

1,2-Dioleoyl-3-trimethylammonium-propane (chloride salt) (DOTAP) was purchased from Avanti Polar Lipids Inc. (Alabaster, AL, U.S.). Cholesterol (Chol) was obtained from Shanghai Bio Life Science & Technology Co., Ltd. (Shanghai, China). MPEG-succinyl-cholesterol conjugate (mPEG-suc-Chol) and folate-PEG-succinyl-cholesterol conjugate (F-PEG-suc-Chol) were synthesized and purified as previously described^55^,^56^. pVax vector was purchased from Life Technologies Corporation (Frederick, MD, U.S.) and served as the negative control. The hTERT promoter regulated plasmid DNA with the vesicular stomatitis virus matrix protein (pMP) constructed according to a previous report^50^. In brief, the cDNA of matrix protein (MP) was amplified by PCR (forward primer, 5′-CGC GGA TCC ATC ATG AGT TCC TTA AAG AAG-3′; reverse primer 5′-CGG AAT TCT CAT TTG AAG TGG CTG ATA GAA TCC-3′), and incorporated a BamHI site at the 5′ end and an EcoRI site at the 3′ end for subcloning into pVax (pVax-MP). A 271-bp fragment containing the core promoter region of hTERT was synthesized and subcloned into the BglI/HindIII-digested pVax-MP plasmid to construct the phTERT-MP plasmid (pMP). The recombinant plasmid pMP was confirmed by restriction endonuclease analysis, PCR, and DNA sequence analysis. The plasmid green fluorescent protein (pGFP) was used in transfection experiments *in vitro*. Plasmid DNA was extracted according to the EndoFree Plasmid Purification Handbook (QIAGEN, Hilden, Germany). Pre-stained protein ladders and DNA ladders were purchased from Fermentas (Thermo Fisher Scientific Inc., Waltham, MA, U.S.). Glucose injection (5%) and sodium chloride injection (0.9%) were obtained from Sichuan Kelun Pharmaceutical Co., Ltd. (Chengdu, Sichuan, China). All other reagents were of analytical grade and were used without further purification except for the chloroform used to prepare liposomes.

### Preparation of FRα-targeted liposomes and lipoplexes

FRα-targeted liposomes (F-LP) were composed of DOTAP, Chol, mPEG-suc-Chol and F-PEG-suc-Chol and prepared by a film dispersion method as previously described^19^. Briefly, DOTAP, Chol, mPEG-suc-Chol and F-PEG-suc-Chol (50:45:4.75:0.25, molar ratio) were dissolved in chloroform. The lipid solution was evaporated on a rotary evaporator to remove the solvent and then the formed thin film was further dried under high vacuum for 6 h. The dry lipid film was hydrated in glucose solution (5%, w/v), and the lipid suspension was sonicated by probe until a translucent lipid suspension (F-P-LP) was obtained. Thereafter, F-P-LP were sterilized through a 0.22-μm microporous membrane (Millipore Ireland BV, Carrigtwohill, Co. Cork, Ireland), and stored at 4 °C until use.

FRα-targeted lipoplexes were prepared by mixing F-LP with pMP or pVax for 30 min at room temperature to formulate F-LP/pMP or F-LP/pVax.

Non-targeted PEGylation liposomes (LP), consisting of DOTAP/Chol/ mPEG-suc-Chol, were prepared by the same method as for F-LP.

Non-targeted PEGylation lipoplexes were prepared by mixing LP with pMP or pVax for 30 min at room temperature to formulate LP/pMP or LP/pVax. All experiments were performed in triplicate.

Liposomes and lipoplexes were photographed with a Canon digital camera (Tokyo, Japan).

### Characterization of liposomes and lipoplexes

The mean particle size, polydispersion index (PDI) and zeta potential of liposomes and lipoplexes were determined with a Zetasizer NanoZS ZEN 3600 (Malvern Instruments, Ltd., Malvern, Worcestershire, U.K.). All experiments were performed in triplicate.

The morphologic characteristics of F-LP and F-LP/pMP were examined with a Tecnai G^2^ F20 transmission electron microscope (TEM, FEI Company, Hillsboro, OR, U.S.).

After lipoplexes were prepared, agarose gel electrophoresis was conducted at pH 7.4 in Tris-acetate-EDTA buffer containing nucleic acid stain Gold View as previously described^19^.

Liposomes, pMP gene and lipoplexes were freeze-dried with a lyophilizer (Boyikang Lab Instrument Co., Ltd., Beijing, China). X-ray photoelectron spectroscopy (XPS, XSAM800, Kratos Analytical Ltd., Manchester, U.K.) was then used to measure the elemental composition of F-LP, and thermogravimetric (TG) and differential thermal analysis (DTA, EXSTAR6000 TG/DTA, Seiko Instruments Inc., Chiba, Japan) were employed to determine the thermal properties of F-LP/pMP.

### Cell culture, *in-vitro* transfection experiments, RT-PCR, and cell cycle and apoptosis analyses

Human ovarian carcinoma cell lines SKOV-3 and A2780 were obtained from the American Type Culture Collection. The cells were cultured as a monolayer in Dulbecco’s Modified Eagles’s Medium supplemented with 10% fetal bovine serum (Hyclone Laboratories Inc., Victoria, Australia) in a humidified atmosphere containing 5% CO_2_ at 37 °C.

*In-vitro* transfection experiments were performed as previously described^55^. F-LP/pGFP and LP/pGFP (containing 1 μg pGFP), were used to transfect SKOV-3 or A2780 cells for 48 h. The transfected SKOV-3 cells were stained with 4′,6-diamidino-2-phenylindole (DAPI) and photographed using an ArrayScan VTI HCS Reader (Thermo Fisher Scientific Inc., Waltham, MA, U.S.). The transfection efficiencies were determined using a FACS Calibur flow cytometer (BD Biosciences, San Jose, CA, U.S.).

About 18 h after SKOV-3 cell transfection with F-LP/pMP, LP/pMP, F-LP/pVax or LP/pVax, total RNA was extracted from each group using an AxyPrep^®^ Multisource Total RNA Miniprep Kit (Axygen Scientific, Inc., Union City, CA, U.S.), and quantified with a NanoDrop 2000 UV-Vis Spectrophotometer (Thermo Fisher Scientific Inc., Waltham, MA, U.S.). Reverse transcription polymerase chain reactions (RT-PCR) were then performed using the PrimeScript^TM^ RT reagent Kit (Takara Biotechnology (Dalian) Co., Ltd., Dalian, Liaoning, China) and 2 × Taq MasterMix kit (Beijing CoWin Biotech Co., Ltd., Beijing, China). The RT-PCR primers for MP gene were 5′-CGC GGA ATC ATG TCC TTA AAG AAG-3′ (forward) and 5′-CGG AAT TCT CAT TGG CTG ATA GAA TCC-3′ (reverse); and the RT-PCR primers for β-actin were 5′-AGA GCT ACG AGC TGC CTG AC-3′ (forward) and 5′-AGC ACT GTG TTG GCG TAC AG-3′ (reverse). pMP and pVax were used as controls^57^.

About 48 h after SKOV-3 cell transfection with F-LP/pMP, LP/pMP, F-LP/pVax, or LP/pVax, total proteins were extracted for western blot analysis of MP expression. The transfected SKOV-3 cells were also stained with propidium iodide for cell cycle and apoptosis analyses, and were examined by flow cytometry.

### *In-vivo* model of advanced ovarian cancer

Athymic female nude mice (BALB/c, 4 to 6 weeks of age, and bred in specific pathogen-free conditions, [SPF]) were purchased from the Vital River (Beijing, China). The mice were then housed and maintained under SPF conditions in our facilities. All experimental protocols involving the mice were in accordance with the Guide for the Care and Use of Laboratory Animals. All procedures were approved and supervised by the State Key Laboratory of Biotherapy Animal Care and Use Committee (Sichuan University, Chengdu, Sichuan, China). The mice were used in studies when they were 6 to 8 weeks old.

Tumor models were established by intraperitoneal (i.p.) injection of SKOV-3 cells (5 × 10^6^ cells in 0.2 mL serum free DMEM) and mice were randomly allocated to six groups^58^,^59^. To assess tumor growth, treatment began seven days after inoculation. Mice were administered every three days with liposomal plasmid DNA i.p. (2.5 μg or 1 μg) in 200 μL of glucose solution (the formulations of lipoplexes and the dosing schedule of gene therapy are shown in Fig. 1 and Supplementary Table S1). The survival group (n = 10 per group) and tumor development group (n = 10 per group) were monitored every three days. Two days after 10 treatments, mice in the tumor development group were anesthetized and sacrificed. Because the peritoneal metastasis model formed many nodules (Fig. 7a), it was difficult to calculate the tumor volume. Therefore, tumor weight was used to evaluate the anti-tumor effects in this study. Whole blood, ascites, tumor tissues, and vital organs were harvested from mice and processed as previously described^19^. In addition, tumor tissues to be used for CD31 staining were immediately placed in McCormick Tissue Freezing Medium OCT, and rapidly frozen for further experimentation.

### Western blot

Total protein concentrations of tumor tissue lysates and the transfected cells were determined with a Bradford protein assay reagent kit (Bio-Rad Laboratories, Hercules, CA, U.S.). MP protein was separated by 10% SDS-PAGE under reducing conditions, and then transferred to Millipore PVDF membranes. Membranes were blocked with 5% skimmed milk and incubated with anti-MP antibody^57^ (Chengdu Zen Bioscience Co., Ltd., Chengdu, Sichuan, China) at 4 °C overnight. Antibody was detected with horseradish peroxidase (HRP)-conjugated secondary antibody and developed with an enhanced chemiluminescence detection kit (Luminnata Crescendo Western HRP Substrate, Millipore Corporation, Billerica, MA, U.S.). β-actin was stained as a control.

### Immunohistochemistry, TUNEL assay and H&E staining

Immunohistochemical analyses of MP expression, Ki_67_ antigen, and microvessel density (MVD, CD31) were carried out with rabbit anti-MP, rabbit anti-human Ki_67_ (Millipore Corporation, Billerica, MA, U.S.), and rabbit anti-mouse CD31 antibodies (Abcam PLC, Boston, MA, U.S.) using the labeled streptavidin-biotin method as previously described^19^. Apoptotic cells in tumor tissues were detected in paraffin sections using a terminal deoxynucleotidyl transferase-mediated dUTP nick end-labeling DeadEnd Fluorometric (TUNEL) system assay kit (Promega, Madison, WI, U.S.) according to the manufacturer’s instructions^19^. The quantification of MVD and TUNEL-positive cells was assessed according to previous reports^19^,^57^,^60^.

The sections were also stained with hematoxylin and eosin (H&E) for histomorphometric analysis. All tissues of three mice in each group were randomly examined with H&E/IHC. All sections were observed or counted by two investigators or pathologists in a blinded fashion.

### Blood tests and serologic biochemical analysis

The samples of whole blood were divided into two parts. One sample was processed with EDTA-2K and directly assayed using a Celltac alpha MEK-6318K fully automatic hematology analyzer (Nihon Kohden Corp., Tokyo, Japan). The other sample was placed at room temperature for 2–3 h and serum was obtained after centrifugation. The serum was then used for serologic biochemical analyses with an automatic analyzer (Hitachi High-Technologies Corp., Tokyo, Japan). Blood tests and serological biochemical analyses were immediately executed once samples were prepared.

### Statistical analyses

Statistical analysis was performed with one-way ANOVA using Statistical Product and Service Solutions software (SPSS V 19.0, IBM Corp., New York, U.S.). When equal variances were assumed after homogeneity of variance test, the Tukey multiple comparisons test was performed. When equal variances were not assumed after homogeneity of variance test, the Tamhane’s T2 multiple comparisons test was used. Differences were considered to be statistically significant at *P* < 0.05. Survivals were estimated using Kaplan-Meier curves and log-rank tests.

## Additional Information

**How to cite this article**: He, Z.-Y. *et al*. Ovarian cancer treatment with a tumor-targeting and gene expression-controllable lipoplex. *Sci. Rep*. **6**, 23764; doi: 10.1038/srep23764 (2016).

## Supplementary Material

Supplementary Information
