# Supplementary material for: Ovarian cancer treatment with a tumor-targeting and gene expression-controllable lipoplex
Source: Sci Rep. 2016 Mar 30;6:23764. doi: 10.1038/srep23764 (PMC4824455; doi:10.1038/srep23764)

Ovarian cancer treatment with a tumor-targeting and gene expression-controllable lipoplex

Zhi-Yao He[1,*], Feng Deng[2,*], Xia-Wei Wei[1], Cui-Cui Ma[1], Min Luo[1], Ping Zhang[1], Ya-Xiong Sang[1,3], Xiao Liang[1], Li Liu[1], Han-Xiao Qin[1], Ya-Li Shen[1,4], Ting Liu[1], Yan-Tong Liu[1], Wei Wang[1], Yan-Jun Wen[1], Xia Zhao[1,2], Xiao-Ning Zhang[5], Zhi-Yong Qian[1] & Yu-Quan Wei[1]

[1] Lab of Aging Research, State Key Laboratory of Biotherapy and Cancer Center, West China Hospital, Sichuan University and Collaborative Innovation Center of Biotherapy, Chengdu, Sichuan 610041, China

[2] Department of Gynecology and Obstetrics, West China Second Hospital, Sichuan University, Chengdu, Sichuan 610041, China

[3] The College of Life Science, Sichuan University, Chengdu, Sichuan 610041, China

[4] Department of Abdominal Oncology, Cancer Center, West China Hospital, Sichuan University, Chengdu, Sichuan 610041, China

[5] Department of Pharmacology and Pharmaceutical Sciences, School of Medicine, Tsinghua University and Collaborative Innovation Center of Biotherapy, Beijing 100084, China

[*] These authors contributed equally to this work.

Correspondence and requests for materials should be addressed to Z.-Y. H. (email: heyaode@163.com) or X.-W. W. (email: xiaweiwei@scu.edu.cn)

Supplementary Table S1. The formulations of lipoplexes and the dosing schedule of gene therapy.

Groups	Formulations (dose for each mouse)	Administration route	Dosing interval
NS	200 μL normal saline	Intraperitoneal	3 days
LP/pVax	200 μL complex containing LP 15 μg and pVax 2.5 μg	Intraperitoneal	3 days
F-LP/pVax	200 μL complex containing F-LP 15 μg and pVax 2.5 μg	Intraperitoneal	3 days
F-LP/$pMP_{(1)}$	200 μL complex containing F-LP 6 μg and pMP 1 μg	Intraperitoneal	3 days
LP/$pMP_{(2.5)}$	200 μL complex containing LP 15 μg and pMP 2.5 μg	Intraperitoneal	3 days
F-LP/$pMP_{(2.5)}$	200 μL complex containing F-LP 15 μg and pMP 2.5 μg	Intraperitoneal	3 days

Supplementary Figure S1. FRα assay and transfection efficiency *in vitro*. a: 95% of SKOV-3 cells overexpress FRα detected by flow cytometry assay (Purple curve: Isotype control. Green curve: Cells were treated with R&D Systems mouse anti-human FOLR1 antibody for 1.5~2 h at 4 ^{o}C, and then the cells were stained with Sigma-Aldrich goat anti-mouse IgG FITC antibody for 30 min at 4 ^{o}C). b. Few A2780 cells (<5%) express FRα by flow cytometry assay (Purple curve: Isotype control. Green curve: Cells were treated with R&D Systems mouse anti-human FOLR1 antibody for 1.5~2 h at 4 ^{o}C, and then the cells were stained with Sigma-Aldrich goat anti-mouse IgG FITC antibody for 30 min at 4 ^{o}C). c: Transfection efficiency of liposomes in A2780 cells (Mean±SD, n=3).

Supplementary Figure S2. H&E staining for liver tissues. The liver cells showed vacuolar degeneration (arrows), as indicated liver cells had been damaged (original magnification, × 200). Whereas, F-LP/pMP$_{(2.5)}$-treated mice showed few vacuolar degeneration of liver cells, and maintained normal liver histology. Because saline did not inhibit the growth and metastasis of cancer cells in abdominal cavity, the livers of mice-treated with saline (NS) were invaded and damaged by ovarian cancer cells. However, F-LP/pMP$_{(2.5)}$ inhibited the growth and metastasis of cancer cells in abdominal cavity, then livers of mice-treated with F-LP/pMP$_{(2.5)}$ were protected and few liver cells with vacuolar degeneration were observed.

Supplementary Figure S3. H&E staining for lung tissues. The inflammatory reaction zone (arrow) of lung treated by F-LP/pMP$_{(2.5)}$ was much less than the lungs of NS, LP/pVax, F-LP/pVax and F-LP/pMP$_{(1)}$ (original magnification, × 200).

Supplementary Figure S4. H&E staining for heart, spleen and kidney. Histological examination results for heart, spleen and kidney were normal by H&E staining (original magnification, × 200).

Supplementary Figure S1.

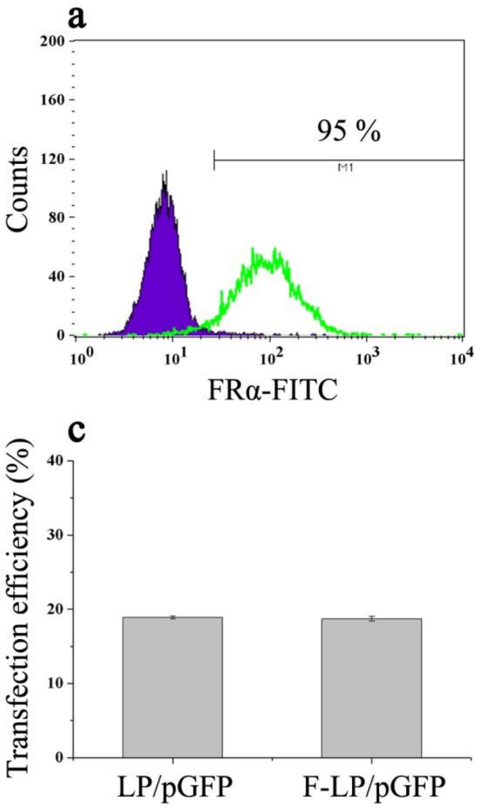

Supplementary Figure S2.

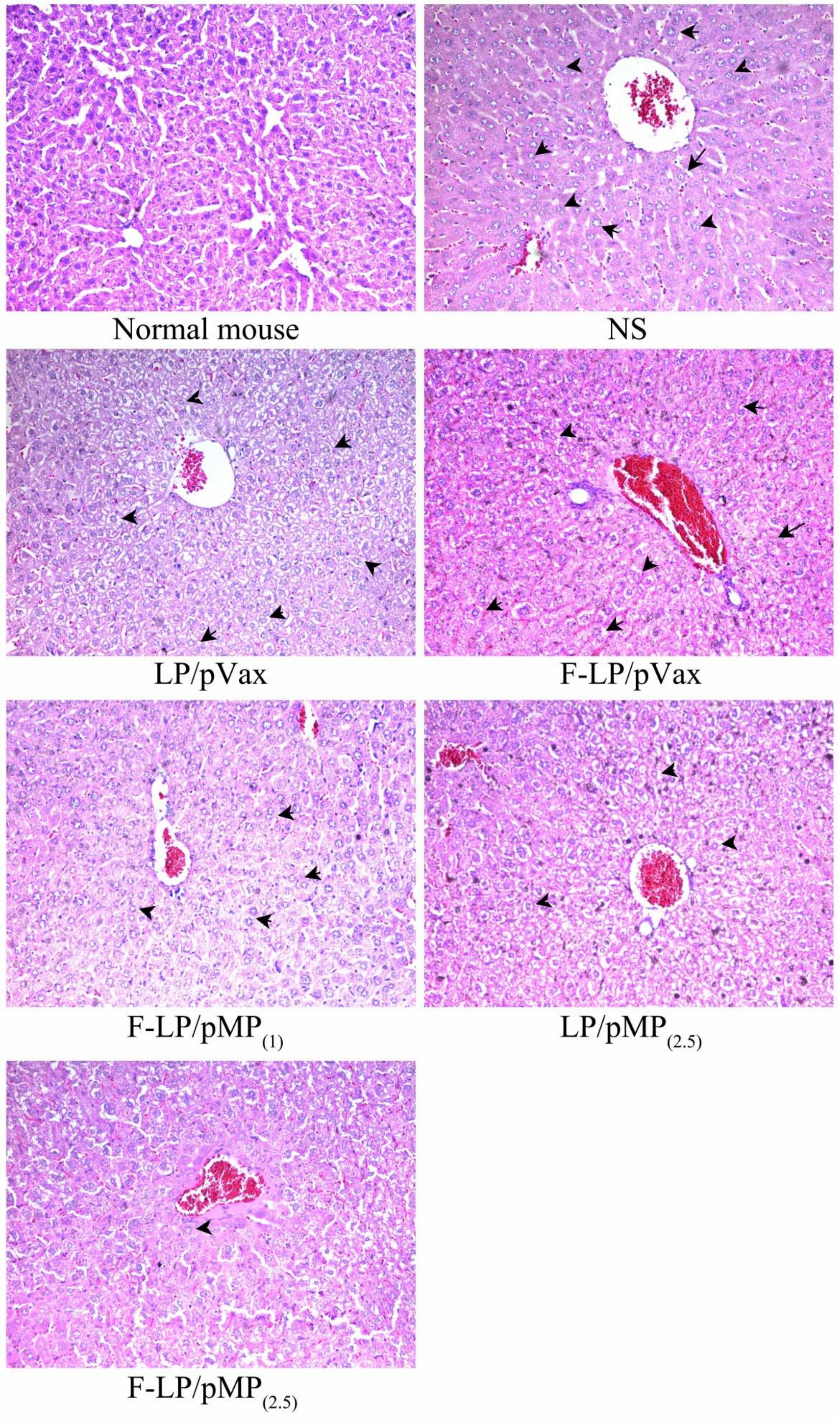

Supplementary Figure S3.

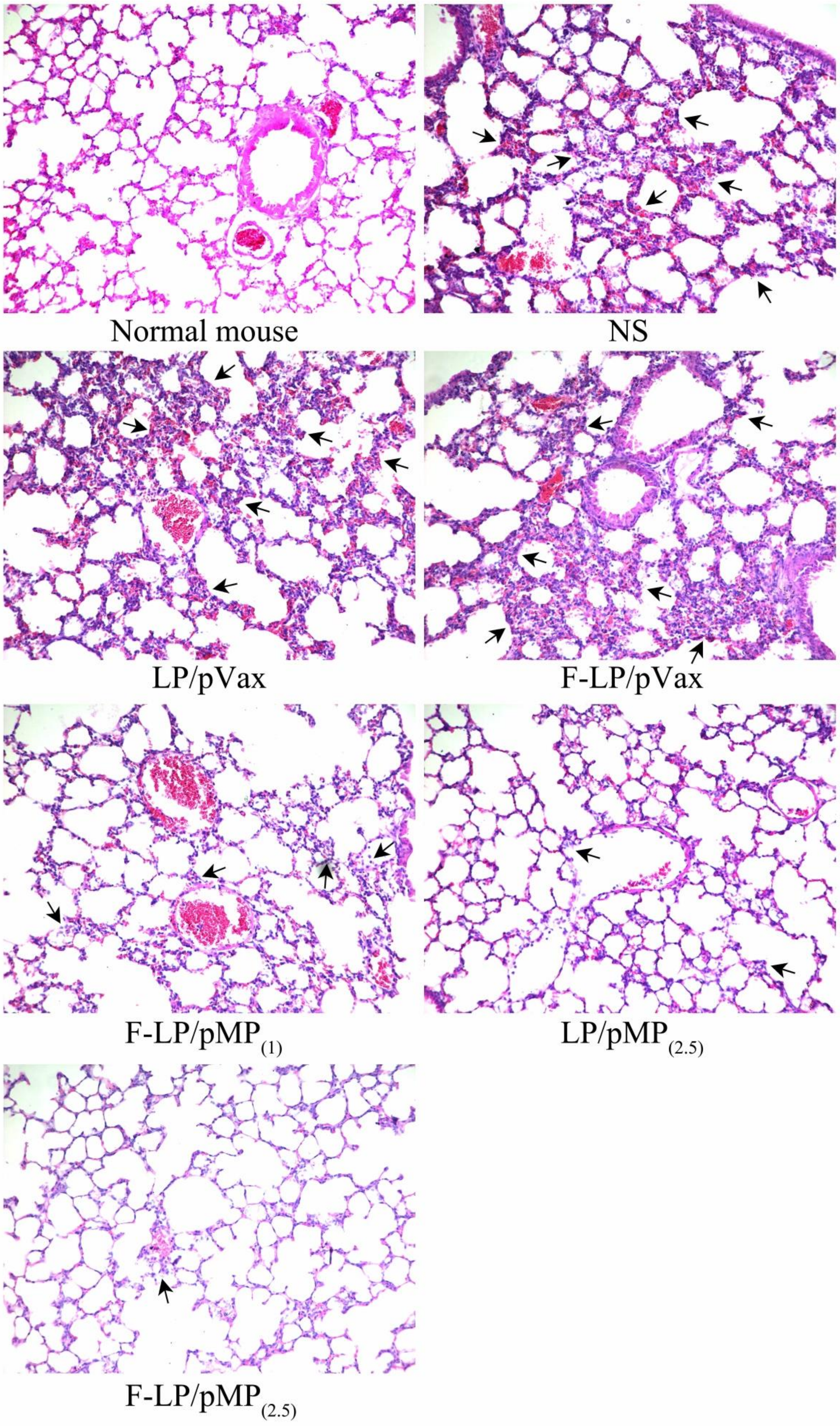

Supplementary Figure S4.

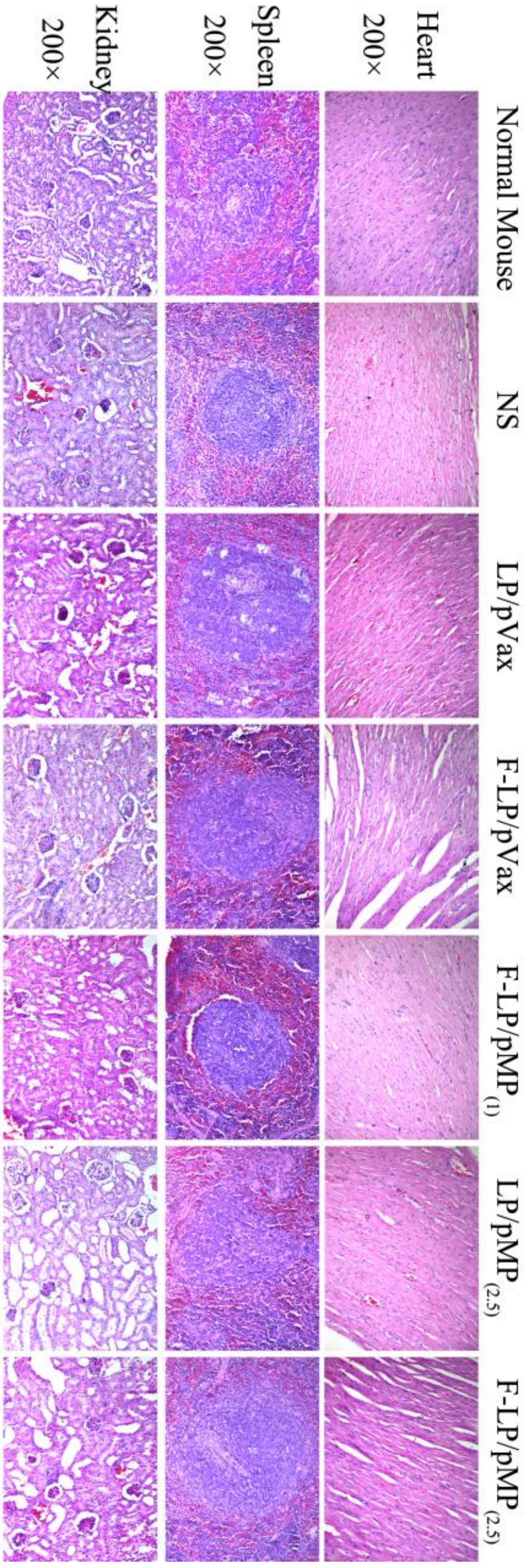

Supplement: Supplementary Information [file srep23764-s1.pdf]
